# A Retinol Derivative Inhibits SARS-CoV-2 Infection by Interrupting Spike-Mediated Cellular Entry

**DOI:** 10.1128/mbio.01485-22

**Published:** 2022-07-13

**Authors:** Liangqin Tong, Lin Wang, Shumin Liao, Xiaoping Xiao, Jing Qu, Chunli Wu, Yibin Zhu, Wanbo Tai, Yanhong Huang, Penghua Wang, Liang Li, Renli Zhang, Ye Xiang, Gong Cheng

**Affiliations:** a Tsinghua-Peking Joint Center for Life Sciences, Beijing Frontier Research Center for Biological Structure and Beijing Advanced Innovation Center for Structural Biology, School of Medicine, Tsinghua University, Beijing, China; b Institute of Infectious Diseases, Shenzhen Bay Laboratory, Shenzhen, Guangdong, China; c Department of Otolaryngology, the Seventh Affiliated Hospital of Sun Yat-sen University, Shenzhen, China; d Department of Thoracic Surgery, the Seventh Affiliated Hospital of Sun Yat-sen University, Shenzhen, China; e Institute of Pathogenic Organisms, Shenzhen Center for Disease Control and Prevention, Shenzhen, Guangdong, China; f Department of Pharmacology, School of Medicine, Southern University of Science and Technology, Shenzhen, China; g Shenzhen Institutes of Advanced Technology, Chinese Academy of Sciences, Shenzhen, Guangdong, China; h Department of Immunology, School of Medicine, the University of Connecticut Health Center, Farmington, Connecticut, USA; Washington University School of Medicine

**Keywords:** SARS-CoV-2, all-*trans* retinoic acid, cellular entry, structural mechanism

## Abstract

Severe acute respiratory syndrome coronavirus 2 (SARS-CoV-2) is the etiological agent of the global pandemic and life-threatening coronavirus disease 2019 (COVID-19). Although vaccines and therapeutic antibodies are available, their efficacy is continuously undermined by rapidly emerging SARS-CoV-2 variants. Here, we found that all-*trans* retinoic acid (ATRA), a vitamin A (retinol) derivative, showed potent antiviral activity against all SARS-CoV-2 variants in both human cell lines and human organoids of the lower respiratory tract. Mechanistically, ATRA directly binds in a deep hydrophobic pocket of the receptor binding domain (RBD) located on the top of the SARS-CoV-2 spike protein (S) trimer. The bound ATRA mediates strong interactions between the “down” RBDs and locks most of the S trimers in an RBD “all-down” and ACE2-inaccessible inhibitory conformation. In summary, our results reveal the pharmacological biotargets and structural mechanism of ATRA and other retinoids in SARS-CoV-2 infection and suggest that ATRA and its derivatives could be potential hit compounds against a broad spectrum of coronaviruses.

## INTRODUCTION

Severe acute respiratory syndrome coronavirus-2 (SARS-CoV-2), the etiological agent of coronavirus disease 2019 (COVID-19), has caused a severe pandemic and public health threat worldwide (1–3). Increasing epidemiological data show that the prevalence, mortality rate, and spread of COVID-19 are rising rapidly. As of 2 February 2022, there have been over 380 million confirmed COVID-19 cases and over 5 million deaths, affecting all countries and territories worldwide (4). SARS-CoV-2 infection causes both mild (approximately 80% of cases) and severe diseases, such as severe pulmonary damage and hyperinflammation. Although several vaccines and therapeutic antibodies are highly effective against early prevalent SARS-CoV-2, their efficacy is compromised by rapidly emerging SARS-CoV-2 variants (5–9). Accordingly, there is an urgent need for the development of new prophylactics and therapeutics, ideally against all SARS-CoV-2 variants and/or a broad spectrum of coronaviruses.

**FIG 1:**
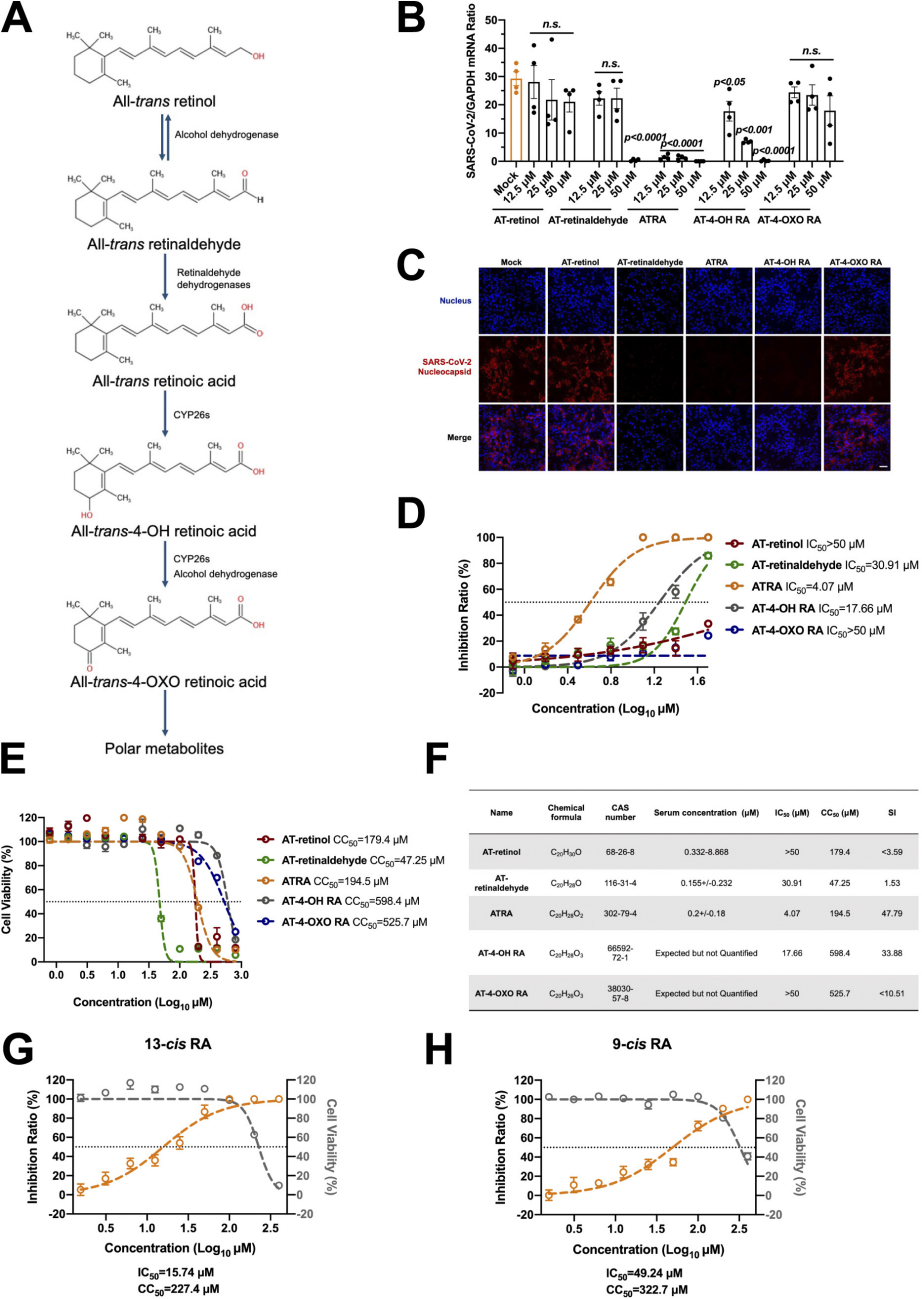
Screening of retinol derivatives against SARS-CoV-2 infection. (A) Vitamin A metabolic pathway with key retinoid metabolite structures and enzymes. CYP26s, cytochrome P450 family. (B and C) Identification of retinol derivates that prevented SARS-CoV-2 infection. (B) Role of 5 retinol derivatives in SARS-CoV-2 infection. Vero cells were incubated with serial concentrations of retinol derivates and then infected with SARS-CoV-2 at an MOI of 0.01. The viral load was detected by qRT-PCR at 48 h postinfection. The data are presented as the mean ± standard error of the mean (SEM). n.s., not significant. The viral load was normalized to human *GAPDH.* The qRT-PCR primers were described in Table S1. (C) Assessment of the antiviral activities of 5 retinol derivates by immunofluorescence staining. Vero cells were incubated with 50 μM compound and then infected with SARS-CoV-2 at an MOI of 0.01. The cells were fixed with 4% PFA at 48 h postinfection. Nucleocapsids were stained with Alexa Fluor 546-conjugated anti-rabbit IgG. The nuclei were stained with DAPI. The stained cells were examined using a Zeiss LSM 880 Meta confocal microscope in multitrack mode. Scale bars, 50 μm. (D to F) Measurement of the half-maximal inhibitory concentration (IC_50_) and cell cytotoxicity (CC_50_) of 5 retinol derivates. (D) Half-maximal inhibitory concentrations of 5 retinol derivates. Vero cells were incubated with gradient concentrations of compounds and then infected with SARS-CoV-2 at an MOI of 0.01. The viral load was detected by a plaque-forming assay at 48 h postinfection. The horizontal dotted line represents the value of the 50% inhibition ratio. (E) Cytotoxicity of 5 retinol derivates in Vero cells. The cells were incubated with gradient concentrations of compounds for 48 h. Cell viability was measured by the Cell Counting Kit-8 (CCK-8) assay. The horizontal dotted line represents the value of 50% cell viability. (F) Comparison of antiviral activity and cytotoxicity of 5 retinol derivates. The selectivity index (SI) is a ratio that compares a drug’s cytotoxicity and antiviral activity: SI = CC_50_/IC_50_. (G and H) Measurement of the IC_50_ (yellow line) and CC_50_ (gray line) of 2 ATRA isomers, 13-*cis* retinoic acid (G) and 9-*cis* retinoic acid (H). Vero cells were incubated with gradient concentrations of compounds and then infected with SARS-CoV-2 at an MOI of 0.01. The viral load was detected by a plaque-forming assay at 48 h postinfection. The cell viability was measured by the CCK-8 assay. The horizontal dotted line represents the value of the 50% inhibition ratio or the value of 50% cell viability. (B to E, G, and H) Experiments were performed independently with at least three biological replicates with comparable results.

The entry of SARS-CoV-2 into host cells is mediated by the virus-encoded glycoprotein spike, which forms trimer spikes on the surface of the virus. The receptor binding domains (RBDs) of the S trimer adapt “up” and “down” two distinct conformations, of which only the “up” RBDs bind the human host receptor protein hACE2 (10–12). Vitamins are essential food supplements and play important roles in the physiological and immune systems (13–15). Vitamin A (retinol) and its derivatives are important to growth, development, reproduction, bone maintenance, epithelial tissue, vision, and normal secretion of mucosal epithelium (16, 17). In addition, vitamin A is a key regulator of various innate and adaptive immune cells and promotes immune homeostasis to interfere with the replication of some RNA viruses (18, 19). Notably, the plasma levels of vitamin A in COVID-19 patients are reduced during acute inflammation, and low levels of vitamin A are closely associated with both acute respiratory distress syndrome and mortality (20), suggesting that vitamin A and its derivatives might be potential bioactive ingredients for a COVID-19 remedy. In animals, vitamin A (retinol) is taken into cells via a cell membrane receptor (21). Subsequently, retinol is oxidized to all-*trans* retinaldehyde (AT-retinaldehyde), which is further metabolized to all-*trans* retinoic acid (ATRA) by retinaldehyde dehydrogenases (22). ATRA can be oxidized to polar metabolites such as all-*trans*-4-OH retinoic acid (AT-4-OH RA) and all-*trans*-4-OXO retinoic acid (AT-4-OXO RA) through cytochrome P450 family members (Fig. 1A).

10.1128/mbio.01485-22.8 TABLE S1 Primers for qRT-PCR. Download Table S1, DOCX file, 0.04 MB. Copyright © 2022 Tong et al. 2022 Tong et al. https://creativecommons.org/licenses/by/4.0/ This content is distributed under the terms of the Creative Commons Attribution 4.0 International license.

## RESULTS

Given the role of retinol and its derivatives in the infection of multiple RNA viruses (18, 23, 24), we assessed the antiviral potency of retinol and its derivatives against SARS-CoV-2. We incubated Vero cells with a serial concentration of retinol and its derivatives and then infected them with SARS-CoV-2. The viral load was quantitated by quantitative RT-PCR (qRT-PCR) at 48 h postinfection. Notably, ATRA, one of the retinol derivatives, presented significant inhibition against SARS-CoV-2 infection (Fig. 1B). The antiviral activity of ATRA was confirmed by immunofluorescence staining of SARS-CoV-2 nucleocapsid in infected cells with a specific monoclonal antibody (Fig. 1C). The 50% inhibitory concentration (IC_50_) of ATRA determined with Vero cells was approximately 4.07 μM (Fig. 1D). Nevertheless, AT-retinaldehyde and AT-4-OH RA, which differs from ATRA in the distal end and cyclohexane head, showed higher IC_50_s of 30.91 μM and 17.66 μM, whereas other metabolites of vitamin A showed only weak anti-SARS-CoV-2 activity in Vero cells (Fig. 1D and E). In light of the selectivity index (SI) calculated by the IC_50_ and 50% cytotoxicity (CC_50_), ATRA had the highest viricidal activity in these retinoids (Fig. 1F). Furthermore, ATRA exhibited higher anti-SARS-CoV-2 activity than its isomers 13-*cis* retinoic acid (13-*cis* RA) (Fig. 1G) and 9-*cis* retinoic acid (9-*cis* RA) (Fig. 1H). Altogether, we chose ATRA to further investigate its role in SARS-CoV-2 infection.

**FIG 2:**
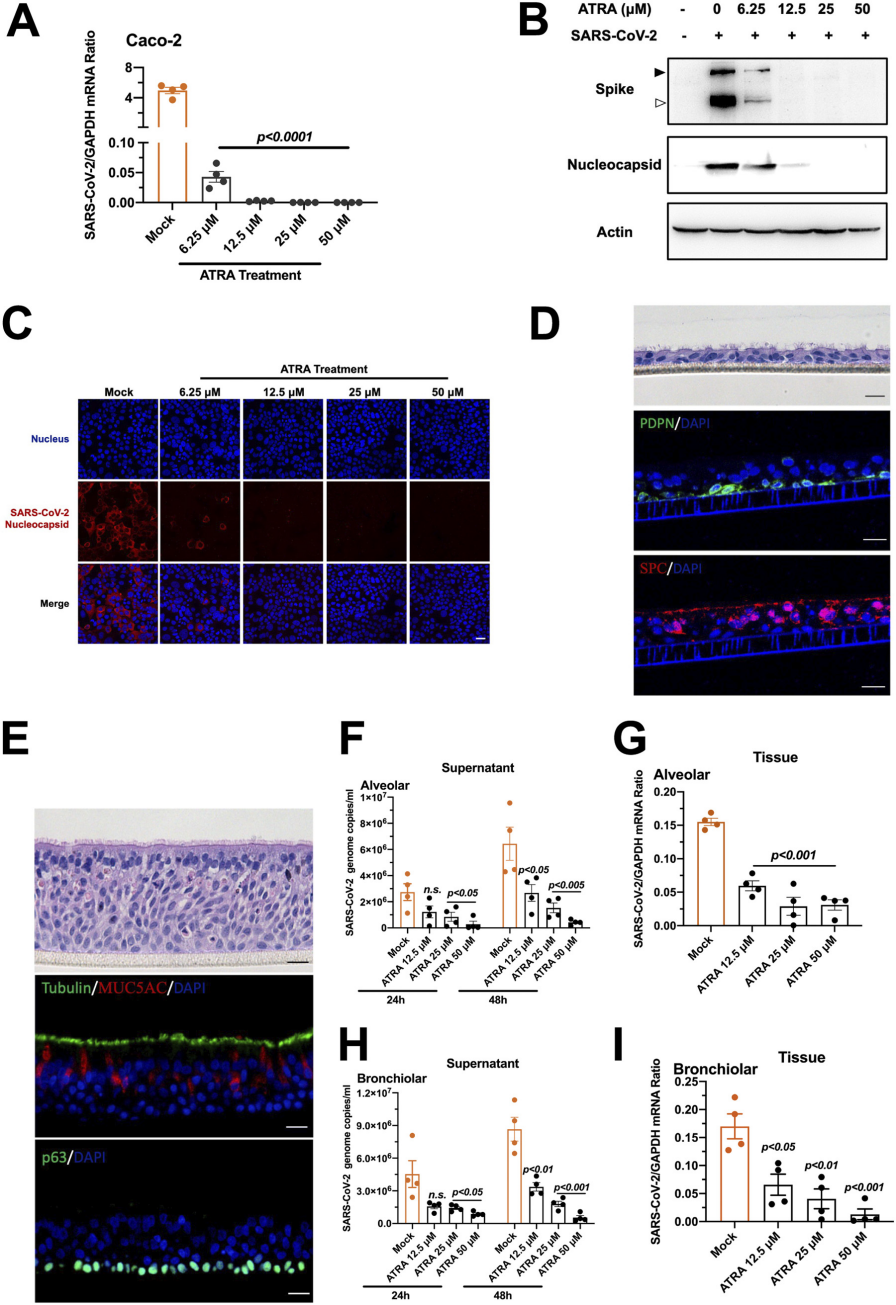
ATRA inhibits SARS-CoV-2 infection in human Caco-2 cells and human respiratory organoids. (A to C) ATRA restricted SARS-CoV-2 infection in human Caco-2 cells. Human Caco-2 cells were incubated with serial concentrations of ATRA and then infected with SARS-CoV-2 at an MOI of 0.01. The viral load was determined by qRT-PCR at 48 h after infection (A). The spike and nucleocapsid proteins in the infected cells were detected by Western blotting (B) and immunofluorescence staining (C). (B) The black arrowhead indicates the full-length spike protein; the white arrowhead indicates the cleaved form. (C) The nucleocapsid was stained with Alexa Fluor 546-conjugated anti-rabbit IgG. The nuclei were stained with DAPI. The stained cells were examined using a Zeiss LSM 880 Meta confocal microscope in multitrack mode. Scale bars, 50 μm. (D to I) Antiviral activity of ATRA in human respiratory organoids. (D and E) Characterization of the human alveolar and bronchiolar organoids. (D) Human alveolar organoids. The differentiated bronchiolar epithelium was indicated by staining with hematoxylin and eosin or specific antibodies labeling type I (PDPN staining) and type II (SPC staining) alveolar epithelial cells. (E) Human bronchiolar organoids. The differentiated alveolar epithelium was composed mainly of ciliated columnar cells (β-IV-tubulin staining), goblet cells (MUC5AC staining), and basal cells (p63 staining). Scale bars, 20 μm. (F to I) Incubation with ATRA reduced SARS-CoV-2 infection in human alveolar (F, G) and bronchiolar (H and I) organoids. Secreted viruses of infected alveolar (F) and bronchiolar (H) organoids were detected in the supernatant at 24 and 48 h postinfection. The viral load in alveolar (G) and bronchiolar (I) organoids was detected at 48 h postinfection. Each dot represents an organoid. The data are presented as the mean ± SEM. The viral load was normalized to human *GAPDH.* The qRT-PCR primers are described in Table S1. (A and F to I) The data were analyzed using an unpaired *t* test. (A to C and F to I) Experiments were performed independently with at least three biological replicates with comparable results.

We next assessed the antiviral effect of ATRA with a human colon carcinoma cell line (Caco-2) (25) and human respiratory organoids that are susceptible to SARS-CoV-2 (26, 27). Consistent with the results from Vero cells, incubation with ATRA reduced the replication of SARS-CoV-2 in a dose-dependent manner in Caco-2 cells (Fig. 2A). This phenotype was further validated by Western blotting (Fig. 2B) and immunofluorescence staining (Fig. 2C). We then tested the antiviral effect of ATRA on human organoids of the lower respiratory tract. Epithelial culture of bronchoscopy specimens was derived from patient biopsy specimens (27), including bronchoscopy and lung parenchymal tissues. The differentiated alveolar epithelium was mainly composed of type I (by podoplanin [PDPN] staining) and type II (by surfactant protein C [SPC] staining) alveolar epithelial cells (Fig. 2D). The differentiated bronchiolar epithelium was detected by staining for a specific surface marker in ciliated columnar cells (β-IV-tubulin staining), goblet cells (MUC5AC staining) and basal cells (p63 staining) (Fig. 2E). Incubation with ATRA reduced the viral load in the human lung alveolar (Fig. 2F and G) and bronchiolar (Fig. 2H and I) organoids in a dose-dependent manner. These data highlighted the potential of ATRA in the control of SARS-CoV-2 infection.

**FIG 3:**
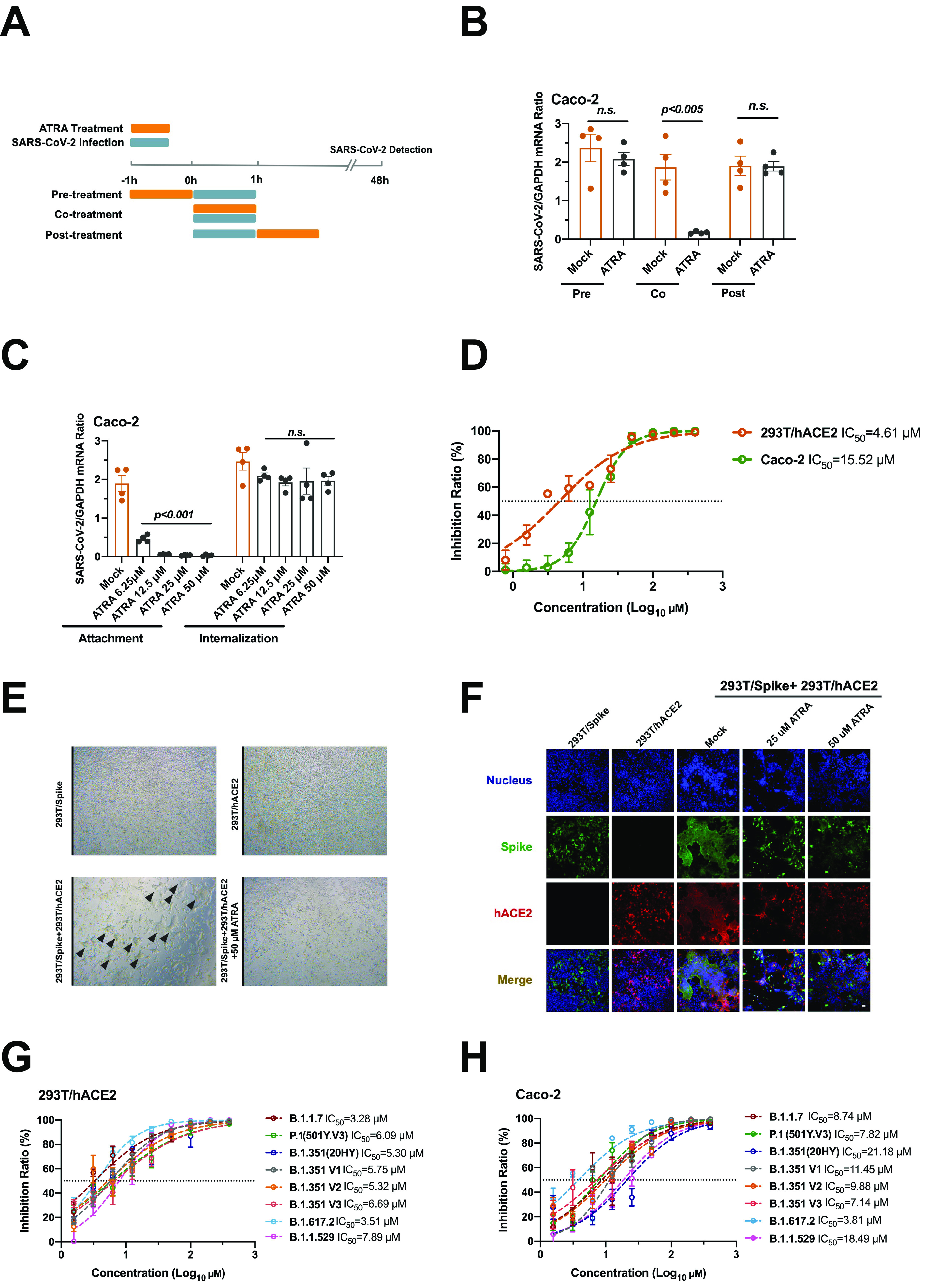
ATRA inhibits SARS-CoV-2 infection by interrupting viral entry. (A to C) ATRA inhibited SARS-CoV-2 attachment to human cells. (A) Schematic diagram of the study design. Human Caco-2 cells were incubated with serial concentrations of ATRA before, during, or after infection. SARS-CoV-2 at an MOI of 0.01 was used to infect the Caco-2 cells. (B) Coincubation of ATRA with the viruses suppressed SARS-CoV-2 infection. (C) Effect of ATRA on the processes of viral attachment to or internalization into Caco-2 cells. For the attachment assay, Caco-2 cells were incubated with a mixture of ATRA and SARS-CoV-2 for 1 h at 4°C, followed by three washes with precooled PBS and incubation for 48 h. For the internalization assay, precooled Caco-2 cells were infected with SARS-CoV-2 at 4°C for 1 h, washed three times with PBS, and then incubated with ATRA at 37°C for 1 h. The cells were then washed and cultured in fresh medium for 48 h for viral burden detection. (B and C) The viral load was determined by qRT-PCR. The data are presented as the mean ± SEM. The viral load was normalized to human *GAPDH.* The qRT-PCR primers are described in Table S1. (D) ATRA inhibited the entry process of SARS-CoV-2 in the pseudovirus luciferase assay. ATRA inhibited spike-mediated viral entry in 293T/hAEC2 (yellow line) and human Caco-2 (green line) cells. An HIV-based pseudovirus system displaying the spike protein (HIV-CoV-2-S) was exploited to assess the role of ATRA in inhibiting the entry of SARS-CoV-2. The effectiveness of SARS-CoV-2 spike-mediated viral entry was determined by a luciferase assay at 48 h postinfection. (E and F) ATRA interrupted spike-ACE2-mediated cell-cell membrane fusion. The 293T cells expressing the SARS-CoV-2 S protein were used as effector cells (293T/Spike); 293T/hACE2 cells expressing human ACE2 were exploited as target cells. The capacity of spike-hACE2-mediated cell-cell fusion was measured by microscopy (E) and immunofluorescence staining (F). (E) The effector cells were aggregated with the target cells to form typical syncytia (black triangles). (F) The spike protein was stained with Alexa Fluor 488-conjugated anti-mouse IgG. Human ACE2 was stained with Alexa Fluor 546-conjugated anti-rabbit IgG. The nuclei were stained with DAPI. The stained cells were examined using a Zeiss LSM 880 Meta confocal microscope in multitrack mode. Scale bars, 20 μm. (G and H) ATRA presented general inhibitory activity against the entry process of various SARS-CoV-2 variants in 293T/hACE2 (G) and human Caco-2 (H) cells. Pseudotyped viruses were generated by the cotransfection of pNL4-3.luc.RE backbone plasmid and pcDNA3.1-SARS-2-S, pcDNA3.1-B.1.1.7-S, pcDNA3.1-P.1(501Y.V3)-S, pcDNA3.1-B.1.351(20HY)-S, pcDNA3.1-B.1.351-V1-S, pcDNA3.1-B.1.351-V2-S, pcDNA3.1-B.1.351-V3-S, pcDNA3.1-B.1.617.2-S, or pcDNA3.1-B.1.1.529-S plasmid to 293T cells. Pseudotyped viruses were incubated with serial dilutions of ATRA and then added to wells containing 293T/hACE2 or Caco-2 cells. The effectiveness of SARS-CoV-2 spike-mediated viral entry was determined by a luciferase assay. (B and C) The data were analyzed using an unpaired *t* test. (B to H) Experiments were performed independently with at least three biological replicates with comparable results.

To assess the infection stage inhibited by ATRA, we treated Caco-2 cells with ATRA 1 h before (pretreatment), simultaneously with (cotreatment), or 1 h after (postinoculation) SARS-CoV-2 inoculation (Fig. 3A). After this treatment, the fresh cell medium was replaced for an additional 48 h of incubation. Both the pretreatment and posttreatment with ATRA showed no effect on the SARS-CoV-2 load. Notably, cotreatment with ATRA and viruses led to a dramatic reduction in the viral load (Fig. 3B), indicating that ATRA may exert its viricidal effect during viral entry. We next assessed the role of ATRA in viral attachment and internalization. SARS-CoV-2 was incubated with Caco-2 cells at 4°C for 1 h in the presence or absence of ATRA to allow viral attachment but not internalization into host cells (28), and then unbound virions were removed by extensive washing with ice-cold phosphate-buffered saline (PBS). The viral load was quantified after stringent washes. The amount of virus attached to the cells treated with ATRA was significantly lower than that of the mock cells, suggesting that ATRA could inhibit the attachment of SARS-CoV-2 to host cells (Fig. 3C, left panel). We next investigated the role of ATRA in viral internalization. We added viruses to Caco-2 cells and incubated them at 4°C for 1 h. Then, the cells were washed with ice-cold PBS and incubated in ATRA-containing medium at 37°C for 1 h. Notably, the SARS-CoV-2 load was not affected by ATRA treatment (Fig. 3C, right panel). The above results suggested a potential role of ATRA in SARS-CoV-2 cellular entry. Consistently, ATRA potently inhibited spike-mediated viral entry in both hACE-2-expressing 293T (293T/hACE2) and human Caco-2 (Fig. 3D) cells in a spike protein-pseudotyped HIV system (HIV-CoV-2-S), further confirming that ATRA directly interrupted spike-mediated SARS-CoV-2 cellular entry.

We next exploited a SARS-CoV-2 S protein-mediated cell-cell fusion assay to assess the role of ATRA during SARS-CoV-2 cellular entry. The 293T cells that express the SARS-CoV-2 S protein were used as effector cells; 293T/hACE2 cells expressing human ACE2 were used as target cells. The effector cells aggregated around a target cell to form a typical syncytium (29). ATRA treatment impaired formation of syncytia between effector cells and target cells. Similarly, spike-hACE2-mediated cell-cell fusion was also significantly affected by ATRA (Fig. 3E and F). HIV-CoV-2-S pseudoviruses were then constructed to assess whether ATRA was still active against various SARS-CoV-2 variants. ATRA reduced the luciferase activity in both 293T/hACE2 (Fig. 3G) and Caco-2 (Fig. 3H) cells infected with all of these HIV-CoV-2-S variants in a dose-dependent manner, suggesting that ATRA was active against SARS-CoV-2 variants.

**FIG 4:**
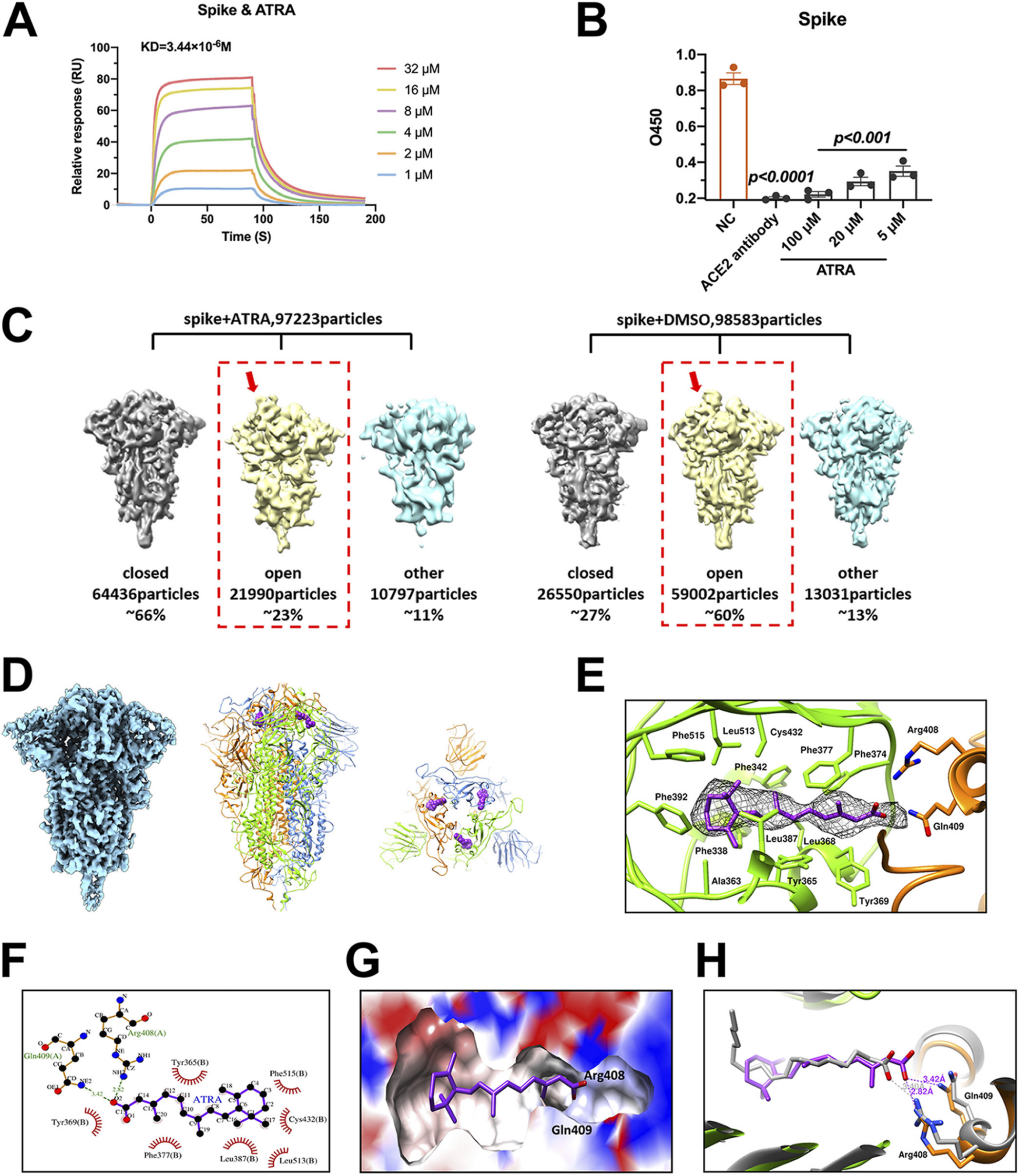
ATRA blocks the interaction between the SARS-CoV-2 spike and human ACE2 proteins by locking all the RBDs in the “down” conformation. (A and B) ATRA directly interacted with the trimeric spike protein to impair the interaction between the SARS-CoV-2 spike and human ACE2 proteins. (A) The interaction of ATRA and SARS-CoV-2 spike protein was detected by SPR. (B) The interaction between the SARS-CoV-2 spike and human ACE2 protein was evaluated with an ELISA kit. About 0.1 μg/well of SARS-CoV-2 spike protein and an hACE2-precoated microplate were used in the assay. The human ACE2 antibody served as a positive control. The data are presented as the mean ± SEM. The data were analyzed using an unpaired *t* test. O450, optical density at 450 nm. C) Heterogenous refinements of spike-ATRA or spike-DMSO particles with previously reported open, closed, and intermediate cryo-EM maps of spike as initial models without symmetry applying (C1) in cryoSPARC; (D, left panel) cryo-EM density map of the closed trimeric spike-ATRA complex with C3 symmetry; (D, middle and right panels) side and top views, respectively, of ribbon representations of the spike-ATRA complex model with the 3 protomers colored blue, green, and orange and with ATRA in purple spheres; (E) additional density in the interface of two adjacent RBDs (shown in green and orange) shown as mesh; (F) ATRA and its interacting amino acids in the binding pocket; (G) ATRA binding pocket shown in surface representation, with positive and negative surface charges colored blue and red, respectively; (H) comparison of ATRA (purple) and LA (gray) binding in the pocket.

We next investigated whether ATRA might block the interaction between the SARS-CoV-2 spike and human ACE2, thereby interrupting viral entry, by using surface plasmon resonance (SPR) and enzyme-linked immunosorbent assay (ELISA). ATRA directly interacted with the trimeric spike protein, with an equilibrium dissociation constant (*K_D_*) of 3.44 × 10^−6^ M (Fig. 4A). Notably, ATRA directly impaired the interaction between the purified trimeric spike and human ACE2 proteins by ELISA (Fig. 4B) and coimmunoprecipitation assays (see Fig. S1A in the supplemental material) in a dose-dependent manner. Nonetheless, ATRA had little effect on the binding of RBD to human ACE2 (Fig. S1B). To explore the mechanism of ATRA-mediated inhibition of the spike-ACE2 interaction, we incubated the purified SARS-CoV-2 spike (30) with 40 μM ATRA or dimethyl sulfoxide (DMSO) as a vehicle control at room temperature for 30 min, identical to the treatment in ELISA, and prepared cryo-electron microscopy (cryo-EM) grids. Heterogenous refinement of the spike-DMSO data in cryoSPARC (31) with previously reported RBD closed, open, and intermediate states of SARS-CoV-2 spike maps (EMD-11203, EMD-11205, and EMD-11206, respectively [30]) as initial models gave a result of ~60% particles in the open state, which had one RBD in the “up” conformation and two RBDs in the “down” conformation. The result was consistent with previous cryo-EM studies on the spike alone (32). In contrast, for the spike-ATRA complex, ~66% of the particles were in the RBD “all-down” closed conformation, whereas only ~23% particles were in the open conformation. This observation gave us a clue that ATRA may inhibit the conformational change of the RBDs from “down” to “up,” which was a prerequisite for the spike-ACE2 interaction (10) (Fig. 4C). The cryo-EM structure of the RBD “all-down” (closed) spike-ATRA complex was determined to a resolution of 3.45 Å with C3 symmetry imposed (Fig. 4D; Fig. S2 and S3 and Table S2). The final refined structure of spike-ATRA was a symmetric homotrimer, of which the receptor ACE2 binding region was partially buried at the contact interface with a neighboring “down” RBD and not fully accessible (Fig. 4D).

10.1128/mbio.01485-22.1 FIG S1 ATRA directly impaired the interaction between human ACE2 protein with the SARS-CoV-2 spike protein but not with RBD. (A) ATRA impaired the interaction between human ACE2 and spike proteins. The interaction between the SARS-CoV-2 spike and human ACE2 was evaluated by coimmunoprecipitation assay. (B) The interaction between the SARS-CoV-2 RBD and human ACE2 was not blocked by incubation with ATRA. The interaction between the SARS-CoV-2 RBD and human ACE2 was evaluated by an ELISA. About 0.1 μg/well of SARS-CoV-2 RBD and an hACE2-precoated microplate were used in the assay. The human ACE2 antibody served as a positive control. The data are presented as the mean ± SEM. The data were analyzed using an unpaired *t* test. Experiments were performed independently with at least three biological replicates with comparable results. Download FIG S1, TIF file, 1.4 MB. Copyright © 2022 Tong et al. 2022 Tong et al. https://creativecommons.org/licenses/by/4.0/ This content is distributed under the terms of the Creative Commons Attribution 4.0 International license.

10.1128/mbio.01485-22.2 FIG S2 Electron microscopy analysis of the ATRA- and DMSO-treated spike protein. The cryo-EM structural analysis pipeline is shown. Download FIG S2, TIF file, 1.0 MB. Copyright © 2022 Tong et al. 2022 Tong et al. https://creativecommons.org/licenses/by/4.0/ This content is distributed under the terms of the Creative Commons Attribution 4.0 International license.

10.1128/mbio.01485-22.3 FIG S3 Cryo-EM structure validation. (A) FSC curves (top) and directional FSC curves (bottom) for the ATRA-treated spike (left panel) and the DMSO-treated spike (right panel) reconstructions with C3 symmetry imposed; (B) cryo-EM density map of the ATRA-spike (left panel) and DMSO-spike (right panel) colored according to local resolution; (C) densities and corresponding models from portions of the N-terminal domain (NTD) (blue), RBD (green), and S2 domain (pink). Residues are shown as sticks, with oxygen atoms colored red and nitrogen atoms blue. The cryo-EM density map is shown as gray mesh. The upper panel shows the ATRA-spike map/model, and the lower panel shows the DMSO-spike map/model. Download FIG S3, TIF file, 1.4 MB. Copyright © 2022 Tong et al. 2022 Tong et al. https://creativecommons.org/licenses/by/4.0/ This content is distributed under the terms of the Creative Commons Attribution 4.0 International license.

10.1128/mbio.01485-22.9 TABLE S2 Cryo-EM data collection, refinement, and validation statistics. Download Table S2, DOCX file, 0.1 MB. Copyright © 2022 Tong et al. 2022 Tong et al. https://creativecommons.org/licenses/by/4.0/ This content is distributed under the terms of the Creative Commons Attribution 4.0 International license.

Additional elongated densities that did not belong to the polypeptide chain could be found near the contact interfaces among the “down” RBDs on top of the trimeric spike protein, which had one distal end embedded in a hydrophobic pocket in one RBD and the other distal end exposed outside the pocket and in contact with a neighboring RBD (Fig. 4E). These densities could only be observed in the closed spike-ATRA C3 map but not in the closed spike-DMSO C3 map, which was also determined to a resolution of 3.51 Å in this study. ATRA could be well fitted to this elongated density, with its cyclohexane head in the pocket and its carboxyl group at the distal tail exposed (Fig. 4E). The cyclohexane head of ATRA in the hydrophobic pocket was surrounded by the side chains of a series of phenylalanines and tyrosines, including Phe338, Phe342, Phe374, Phe377, Phe392, and Phe515 and Tyr365 and Tyr369 (Fig. 4F and G). Incorporation of any polar group into the cyclohexane head of ATRA, as with the downstream metabolites AT-4-OH RA and AT-4-OXO RA, would disrupt the hydrophobic interactions and dramatically reduce the binding affinity to the spike (Fig. S4A and B). Arg408 and Gln409 from a neighboring RBD form a fork firmly holding the carboxyl tail of the bound ATRA through salt bridges and hydrogen bonds, which hinder the movement of the RBD and lock the RBDs in an all “down” conformation, thus preventing their interaction with ACE2. The results also clearly explained why ATRA had no effect on interrupting the interactions between the purified RBD and ACE2, as shown in the ELISA, in which no interfaces existed among the free RBDs. Distances between the gravity centers of the “down” RBDs in the ATRA-S complex were 31.9 Å, which was much shorter than the distances (34.4 Å) measured between the “down” RBDs in the DMSO-treated-spike’s structure (Fig. S5), suggesting that the bound ATRA significantly enhanced the interactions between the “down” RBDs. In addition, ATRA binding also induced an ~3° anticlockwise rotation of the “down” RBDs in the ATRA-S complex relative to those in the DMSO-treated spikes (Fig. S5).

10.1128/mbio.01485-22.4 FIG S4 Binding affinity of retinol derivatives with the SARS-CoV-2 spike protein. The interaction between the SARS-CoV-2 spike and AT-4-OH retinoic acid (A), AT-4-OXO retinoic acid (B), AT-retinol (C), and AT-retinaldehyde (D) was determined by the SPR assay. The concentrations of chemicals that flowed over the chip surface ranged from 1 to 32 μM. The binding kinetics and affinity were analyzed by using the Biacore Insight Evaluation software (GE Healthcare). Download FIG S4, TIF file, 1.4 MB. Copyright © 2022 Tong et al. 2022 Tong et al. https://creativecommons.org/licenses/by/4.0/ This content is distributed under the terms of the Creative Commons Attribution 4.0 International license.

10.1128/mbio.01485-22.5 FIG S5 Structural comparison of ATRA- and DMSO-treated spike structures. (A, left panel) Superposition of S protomers of the ATRA-treated spike (in purple) and the DMSO-treated spike (in dim gray) shows consistency of the S2 domain but discrepancy in the RBD domain. (A, right panel) The S2 domain and RBD are illustrated as axes for comparison. (B) Orientation comparison of the RBD domains in the ATRA- and DMSO-treated spike structure; (C) measurements of the distances between centroids of RBDs. The relative rotation angle between the RBDs is indicated. Download FIG S5, TIF file, 1.6 MB. Copyright © 2022 Tong et al. 2022 Tong et al. https://creativecommons.org/licenses/by/4.0/ This content is distributed under the terms of the Creative Commons Attribution 4.0 International license.

Retinol and AT-retinaldehyde had the same head and stem structure as ATRA, but differ only in the tail, of which retinol and AT-retinaldehyde had a hydroxyl group and a carbonyl group, respectively. Given that retinol and AT-retinaldehyde have much weaker binding affinity (Fig. S4C and D) to spike than the binding affinity of ATRA (*K_D_* = 3.44 × 10^−6^ M), the carboxyl tail of the bound ATRA was essential for establishing strong interactions with neighboring RBDs. The RBDs of native spike proteins were supposed to be in a dynamic transformation between the “up” and “down” conformations. Once binding to the pocket, ATRA functioned like a bolt to lock the spike protein in a compact closed state and was unable to interact with ACE2.

To further validate the structural model, we aimed to generate the spike proteins with single mutation at Arg408 to Ala (R408A) and Gln409 to Ala (Q409A), as well as the R408A Q409A double mutation. The mutants were expressed in HEK293F cells and purified with StrepTactin resins by following the same routine as for the wild-type S. Nonetheless, only the R408A mutant was expressed and purified successfully, while no expression was detected for either the Q409A or R408A Q409A mutant, suggesting that Q409 may play an essential role in folding and assembly of the spike (Fig. S6A). The competition ELISA result revealed that ATRA does not impair the interaction between the R408A mutant and hACE2, confirming that the carboxyl tail of ATRA-mediated interactions between the RBDs is critical for blocking the binding of hACE2 (Fig. S6B). In addition, we assessed the interaction between the R408A mutant and ATRA in the SPR assay, and the purified RBD monomer protein was served as a control (Fig. S6C and D). We found that neither R408A protein nor RBD monomer can bind to ATRA, indicating that the neighboring RBD is essential for the binding and inhibition effect of ATRA. The combined results suggested that ATRA inhibits the interaction of spike trimer protein with hACE2 through hindering the “up” movement of the RBDs.

10.1128/mbio.01485-22.6 FIG S6 ATRA did not impair the interaction between mutant spike R408A and human ACE2. (A) SDS-PAGE analysis of the elution from StrepTactin resins. R408A, Q409A, and R408A Q409A (double mutation [DM]) mutants were expressed in HEK293F cells and captured by StrepTactin resins. The proteins captured by StrepTactin resins were then eluted with 2.5 mM desthiobiotin. No expression was detected for both Q409A and DM proteins. Only the R408A protein was detected by SDS-PAGE. (B) The interaction between the R408A mutant protein and human ACE2 protein was evaluated by an ELISA. Approximately 0.1 μg/well of R408A mutant protein and a microplate precoated with hACE2 were used in the assay. The human ACE2 antibody served as a positive control. The data are presented as the mean ± SEM. The data were analyzed using an unpaired *t* test. Binding affinity of ATRA with R408A mutant protein and the RBD monomer is shown. The interaction between the ATRA and R408A mutant protein (C) and RBD monomer (D) was determined by the SPR assay. The concentrations of ATRA that flowed over the chip surface ranged from 1 to 32 μM. The binding kinetics and affinity were analyzed by using the Biacore Insight Evaluation software (GE Healthcare). Download FIG S6, TIF file, 1.8 MB. Copyright © 2022 Tong et al. 2022 Tong et al. https://creativecommons.org/licenses/by/4.0/ This content is distributed under the terms of the Creative Commons Attribution 4.0 International license.

Interestingly, linoleic acid (LA) was found to bind the same pocket of the spike proteins of SARS-CoV-2 and bat and pangolin coronaviruses in high-resolution cryo-EM structures (33–35). The bound LA reported previously was naturally incorporated into the spikes during recombinant production of the spikes and presented largely an “L” shape in the pocket with a short carboxyl tail exposed. The short carboxyl tail is oriented far away from residues Arg408 and Gln409 of the neighboring RBD, leading to only weak van der Waals interactions with the neighboring RBD (Fig. 4H). Thus, a significant portion of the RBD “up” conformation could be observed in the spike-LA study (33). Intriguingly, the binding pocket is also conserved in SARS-CoV and Middle East respiratory syndrome coronavirus (MERS-CoV) (33). We next assessed whether ATRA inhibits the infection of other coronaviruses using luciferase assays. The results showed that ATRA administration impaired the entry of HIV-SARS-CoV-S pseudovirus into 293T/hACE2 cells and significantly reduced the infection of HIV-MERS-CoV-S into Huh7 cells (Fig. S7A) (29). Nonetheless, incubation with ATRA did not impact infection with the influenza A virus (IAV) H1N1 WSN strain in A549 cells (Fig. S7B), suggesting that the antiviral activity of ATRA was specific to coronaviruses. Such an LA- or ATRA-binding pocket has been identified or predicted in the spike proteins of several human, bat, and pangolin coronaviruses, suggesting evolutionary conservation of this site, which could be an attractive pancoronavirus drug target.

10.1128/mbio.01485-22.7 FIG S7 ATRA inhibited the infection of other coronaviruses. (A) ATRA presented general inhibitory activity against the entry process of SARS-CoV in 293T/hACE2 cells (green line) and MERS-CoV in Huh7 cells (blue line). The effectiveness of spike-mediated viral entry was determined by a luciferase assay. The gray dotted line represents the value of the 50% inhibition rate. The data are presented as the mean ± SEM. (B) Incubation with ATRA did not inhibit the infection of the IAV H1N1 WSN strain in A549 cells. The amount of viral RNA was normalized to human *GAPDH*. The data are presented as the mean ± SEM. The data were analyzed using an unpaired *t* test. Experiments were performed independently with at least three biological replicates with comparable results. Download FIG S7, TIF file, 1.2 MB. Copyright © 2022 Tong et al. 2022 Tong et al. https://creativecommons.org/licenses/by/4.0/ This content is distributed under the terms of the Creative Commons Attribution 4.0 International license.

## DISCUSSION

Retinoids, a group of compounds including vitamin A and its active metabolite ATRA, regulate serial physiological activity in multiple organ systems, such as cell growth, differentiation, and apoptosis (36–38). Indeed, differentiation therapy with ATRA has marked a major advance, and ATRA has become the first-choice drug in the treatment of acute promyelocytic leukemia (39–42). In addition, ATRA and its isomers have also been successfully applied to a variety of dermatological conditions, such as psoriasis, acne, and ichthyosis (43–46). Given a large clinical potential, the synthesis of ATRA analogues with high efficiency has attracted great attention. Indeed, the ATRA analogues reported to date include more than 4,000 natural and synthetic molecules that are structurally and/or functionally related to ATRA (47). These clinical applications suggest the pharmacological feasibility of using ATRA or its derivatives for the remedy and prevention of COVID-19 disease. In this study, we found that ATRA showed potent antiviral activity against SARS-CoV-2 infection in both human cell lines and human organoids of the lower respiratory tract. ATRA blocked the interaction between the SARS-CoV-2 spike and human ACE2 proteins by locking the RBDs in “all-down” conformations on the spike trimer and thus inhibits its interaction with ACE2. The interruption of receptor-ligand interactions impaired the fusion between the SARS-CoV-2 envelope and the host cell membrane, thereby inhibiting SARS-CoV-2 spike-mediated cellular entry. Here, we reveal the pharmacological biotargets, function, and structural mechanism of ATRA and other retinoids in SARS-CoV-2 infection, thus suggesting that treatment with ATRA or its derivatives may act as a potent clinical remedy option for COVID-19 diseases. Considering the conservation of the ATRA-binding pocket in the spike protein of several coronaviruses, it is worth exploring ATRA and developing new ATRA analogues as potential antipancoronavirus hit compounds.

## MATERIALS AND METHODS

### Ethics statement.

Human airway biopsy specimens were collected with written informed consent from pulmonary bulla patients with approval of the local ethics committee at Shenzhen Institutes of Advanced Technology, Chinese Academy of Sciences, and No. 7 Affiliated Hospital of Zhongshan University.

### Cell lines, viruses, plasmids, and peptides.

Vero, Caco-2, 293T, and MDCK cells were purchased from ATCC (catalog no. CCL81, HTB37, CRL3216, and CCL-34), cultured in Dulbecco’s modified Eagle’s medium (DMEM) (Gibco catalog no. 11965-092) supplemented with 10% heat-inactivated fetal bovine serum (FBS) (Gibco catalog no. 16000-044), and 1% antibiotic-antimycotic (Gibco catalog no. 15240-062) at 37°C and 5% CO_2_. The 293T cells expressing human ACE2 (293T/hACE2) were a gift from Qiang Ding from Tsinghua University and were cultured under the same conditions. SARS-CoV-2 (GISAID accession no. EPI_ISL_413879) was propagated in Vero cells and titrated by a standard plaque-forming assay followed by storage of aliquots at −80°C until further use in the experiments. Influenza A virus (IAV) H1N1 A/WSN/33 strain was passaged in MDCK cells cultured in DMEM with 0.3% FBS and 0.5 mg/mL TPCK (*N*-p-tosyl-phenylalanine chloromethyl ketone)-treated trypsin (Sigma catalog no. T1426). Plasmids expressing the SARS-CoV-2 wild-type spike protein (pcDNA3.1-SARS-2-S) were kindly provided by Lu Lu from Fudan University. Plasmids expressing the spike protein of SARS-CoV-2 variants pcDNA3.1-B.1.1.7-S, pcDNA3.1-P.1(501Y. V3)-S, pcDNA3.1-B.1.351(20HY)-S, pcDNA3.1-B.1.351-V1-S, pcDNA3.1-B.1.351-V2-S, pcDNA3.1-B.1.351-V3-S, pcDNA3.1-B.1.617.2-S, pcDNA3.1-B.1.1.529-S, and pNL4-3.luc.RE (the luciferase reporter-expressing HIV-1 backbone) was maintained in our laboratory. Plasmids expressing the spike protein of SARS-CoV and MERS-CoV were kindly provided by Linqin Zhang from Tsinghua University.

### Antiviral activity assay.

Compounds were dissolved in dimethyl sulfoxide (DMSO) and subsequently diluted with cell culture medium for cell incubation. Vero cells were preincubated with serial concentrations of each compound at 37°C for 1 h and then infected with SARS-CoV-2 at a multiplicity of infection (MOI) of 0.01 for 1 h. After three washes with PBS, cells were cultured with compound-containing medium for an additional 48 h. Vero cells were harvested, and the viral burden was detected by qRT-PCR.

### RNA extraction and qRT-PCR.

Total RNA was isolated from infected cells using a Multisource RNA miniprep kit (Axygen catalog no. AP-MN-MS-RNA-250) and reverse transcribed to cDNA using an iScript cDNA synthesis kit (Bio-Rad catalog no. 1708890). The viral burden was quantified with qRT-PCR using iTaq Universal SYBR green supermix (Bio-Rad catalog no. 1725121) on the Bio-Rad CFX-96 Touch real-time detection system. Primer sequences are shown in Table S1 in the supplemental material. The amount of virus was normalized to the human *GAPDH* gene (GenBank accession no. NC_000012.12).

### Immunofluorescence staining and microscopy.

For the analysis of the antiviral activity of compounds, cells were preincubated with compounds, infected, and incubated for 48 h prior to immunofluorescence staining. For the cell-cell fusion assay, 293T/hACE2 cells were incubated with 293T cells transfected with the SARS-CoV-2 spike protein in the presence or absence of ATRA. The cocultured cells were incubated for 24 h, and the state of cell fusion was observed by microscopy and immunofluorescence staining.

Cells were washed three times in PBS and fixed with 4% paraformaldehyde (PFA) for 2 h at room temperature. Then, the cells were permeabilized with 0.1% Triton X-100 for 30 min, washed with PBS, blocked with 2% bovine serum albumin (BSA) for 1 h, and stained with anti-SARS-CoV-2 nucleocapsid (Abcam catalog no. ab271180), anti-SARS-CoV-2 spike (Abcam catalog no. ab273433), and anti-hACE2 (Abcam catalog no. ab108252) antibodies for 2 h at room temperature, according to different experimental settings. The cells were washed with PBS and then stained with secondary antibodies for 1 h at room temperature. The nuclei were stained with DAPI (4′,6-diamidino-2-phenylindole) (Abcam catalog no. ab228549). Images were examined by a Zeiss LSM 880 Meta confocal microscope.

### IC_50_ quantification.

Vero cells were seeded in a 48-well plate at a density of 2 × 10^4^ cells per well and incubated for 12 h before the experiment. Compounds were diluted with cell culture medium at concentrations ranging from 0.78 to 50 μM and preincubated with cells at 37°C for 1 h. Cells were infected with SARS-CoV-2 at an MOI of 0.01 for 1 h, washed three times with PBS, and cultured with compound-containing medium for 48 h. The viral titer in the supernatant was determined by a plaque-forming assay. The concentration of each compound required to inhibit viral infection by 50% (IC_50_) was calculated by comparing the values with the DMSO-treated cells using the dose-response inhibition model in GraphPad Prism 8.0 (GraphPad Software, USA).

### Cell viability.

Cell viability was estimated by the Cell Counting Kit-8 (CCK-8) (Meilunbio catalog no. MA0218-1). Briefly, 10,000 Vero cells were seeded in a 96-well plate, incubated at 37°C overnight, and then administered gradient concentrations of compounds at 37°C for 48 h. The CCK-8 solution was added, followed by an additional 1-h incubation at 37°C. Absorbance was measured at 450 nm. The concentration of each compound necessary to reduce cell viability by 50% (CC_50_) was calculated by comparing the values with DMSO-treated cells using a sigmoidal nonlinear regression function in GraphPad Prism 8.0 (GraphPad Software, USA).

### Western blot analysis.

Caco-2 cells were seeded in a 6-well plate at a density of 5 × 10^5^ cells per well and incubated for 12 h before the experiment. Cells were preincubated with ATRA at 37°C for 1 h, infected with SARS-CoV-2 at an MOI of 0.01 for 1 h, washed three times with PBS, and cultured in ATRA-containing medium for 48 h. Cells were harvested and denatured in protein loading buffer at 100°C for 10 min for Western blot analysis. The following antibodies were used in these experiments: anti-SARS-CoV-2 spike (Abcam catalog no. ab273433), anti-SARS-CoV-2 nucleocapsid (Abcam catalog no. ab271180), anti-hACE2 (Abcam catalog no. ab108252), and antiactin (Cell Signaling Technology catalog no. 3700).

### Air-liquid interface human epithelial cultures and viral infection.

Air-liquid interface (ALI) human epithelial cultures were kindly provided by Liang Li from the Shenzhen Institutes of Advanced Technology, Chinese Academy of Sciences. ALI human epithelial cultures were used to evaluate antiviral activity at day 28. Briefly, ALI cultures were preincubated with ATRA at 37°C for 30 min, infected with 5,000 PFU of SARS-CoV-2, and incubated at 37°C for 1 h. The inoculum was removed, the cultures were washed three times with PBS, and ATRA was added to the Transwell for further incubation. At 24 h and 48 h postinfection, 150 μL of PBS was added to the top surface of ALI cultures, and the cells were then harvested for viral genome copy determination. The epithelia were harvested for viral burden quantification at 48 h postinfection.

### Histological analysis and multiplex immunofluorescence staining of ALI cultures.

ALI cultures were fixed in 4% PFA, and paraffin sections (3 μm thick) were prepared routinely. Sections were treated with xylene and a graded series of alcohol solutions and incubated in citrate buffer (pH 6.0) and microwaved at 95°C for 20 min for antigen retrieval. Sections were stained with hematoxylin and eosin for histological analysis. The NEON 7-color Allround Discovery kit for formalin-fixed, paraffin-embedded (FFPE) sections (Histova Biotechnology catalog no. NEFP750) was used for multiplex fluorescence labeling. Briefly, sections were quenched for endogenous peroxidases in 3% H_2_O_2_ in methanol for 20 min. After undergoing blocking for 30 min at room temperature, the sections were incubated with the primary antibody for 2 h at 37°C, followed by detection using a horseradish peroxidase (HRP)-conjugated secondary antibody and TSA-dendron-based fluorophores. Subsequently, the sections were incubated in retrieval/elution buffer at 95°C for 10 s to eliminate the primary and secondary antibodies. The following antibodies were used in this study: anti-β-IV-tubulin (Abcam catalog no. ab179504), anti-MUC5AC (Abcam catalog no. ab198294), anti-p63 (Abcam catalog no. ab53039), antipodoplanin (Abcam catalog no. ab236529), and anti-SPC (Novusbio catalog no. NBP1-87201). After all the antibodies were detected sequentially, the epithelial sections were stained with DAPI (Abcam catalog no. ab228549) and cleared by the clearing-enhanced three-dimensional (3D) method. Images were captured by a Zeiss LSM 880 Meta confocal microscope.

### Time course inhibition assay.

To estimate the impact of ATRA on the SARS-CoV-2 replication cycle, Caco-2 cells were infected at an MOI of 0.01 for 1 h at 37°C. ATRA was added 1 h before (pretreatment), simultaneously with (cotreatment), or 1 h after (posttreatment) SARS-CoV-2 inoculation. For the pretreatment assay, cells were first incubated with ATRA at 37°C for 1 h, followed by three washes with PBS, and then infected with SARS-CoV-2 for 1 h. For the cotreatment assay, cells were simultaneously incubated with SARS-CoV-2 and ATRA for 1 h at 37°C. Then, the mixture was removed, and the cells were washed three times with PBS before fresh medium was added. For the posttreatment assay, cells were infected with SARS-CoV-2 for 1 h, washed three times with PBS, and then incubated with ATRA-containing medium for 1 h. At 48 h postinfection, cells were harvested for viral burden detection by qRT-PCR.

To determine the specific stage of the viral entry process affected by ATRA, a time-of-addition assay was performed. Briefly, for the attachment assay, Caco-2 cells were precooled on ice and then incubated with a mixture of ATRA and SARS-CoV-2 for 1 h at 4°C, followed by three washes with precooled PBS and incubation at 37°C for 48 h for viral burden detection. For the internalization assay, precooled Caco-2 cells were infected with SARS-CoV-2 at 4°C for 1 h, washed three times with PBS, and then incubated with ATRA at 37°C for 1 h. The cells were then washed and cultured in fresh medium for 48 h for viral burden detection.

### Inhibition of pseudotyped SARS-CoV-2 and variant infection.

Pseudotyped viruses were generated by the cotransfection of pNL4-3.luc.RE backbone plasmid and pcDNA3.1-SARS-2-S, pcDNA3.1-B.1.1.7-S, pcDNA3.1-P.1(501Y.V3)-S, pcDNA3.1-B.1.351(20HY)-S, pcDNA3.1-B.1.351-V1-S, pcDNA3.1-B.1.351-V2-S, pcDNA3.1-B.1.351-V3-S, pcDNA3.1-B.1.617.2-S, pcDNA3.1-B.1.1.529-S, pcDNA3.1-SARS-S, or pcDNA3.1-MERS-S plasmid to HEK293T cells. The medium was changed 12 h after transfection. At 48 h after transfection, the supernatant was harvested, centrifuged at 3,000 × *g* for 10 min, and frozen at −80°C until further use in the experiments. Pseudotyped viruses were incubated with serial dilutions of ATRA for 30 min at 37°C, and then 40,000 Caco-2 or HEK293T/hACE2 cells were added to each well. Plates were incubated at 37°C for 48 h, washed with PBS, and lysed with 1× passive lysis buffer (Promega catalog no. E1941). Luciferase activity was analyzed using a luciferase assay system (Promega catalog no. E1910).

### Protein expression and purification.

For expression of the prefusion S ectodomain, a gene encoding residues 1 to 1208 of SARS-CoV-2 (GenBank accession no. MN908947) spike with proline substitutions at residues 986 and 987, a “GSAS” substitution at the furin cleavage site (residues 682 to 685), a C-terminal trimerization motif, an HRV3C protease cleavage site, and a Twin-Strep-tag were synthesized and cloned into the mammalian expression vector pCMV. For mutant plasmid construction, R408, Q409, and R408 Q409 were substituted with alanine. The constructed plasmid was confirmed by sequencing and used in transient transfection of HEK293F cells with polyethylenimine. At 48 h posttransfection, protein was purified from filtered and buffer-exchanged cell culture medium using StrepTactin resin (IBA). The eluted protein was further purified by size exclusion chromatography (SEC) with a Superose 6 10/300 column (GE Healthcare) in 20 mM HEPES (150 mM NaCl [pH 7.5]). Peak fractions from SEC were analyzed by SDS-PAGE, flash frozen with liquid nitrogen, and stored at −80°C until further use.

### Surface plasmon resonance assay.

The purified SARS-CoV-2 spike protein was captured on a CM5 sensor chip (GE Healthcare catalog no. BR100530) with approximately 8,000 response units in the test flow channels, and a blank channel was employed as a negative control. The SPR experiments were performed using a Biacore 8K system at 25°C with a flow rate of 30 μL/min in buffer A (101 mM Na_2_HPO_4_, 18 mM KH_2_PO_4_, 27 mM KCl, 1.37 M NaCl, 5% DMSO, 0.05% Tween 20 [pH 7.4]). Chemicals were dissolved and diluted with buffer A. Concentrations of chemicals ranging from 1 μM to 32 μM flowed over the chip surface. The binding kinetics and affinity were analyzed using Biacore Insight Evaluation software (GE Healthcare).

### Enzyme-linked immunosorbent assay.

ELISA was performed by a SARS-CoV-2 surrogate virus neutralization test kit (GenScript catalog no. L00847), which contained an hACE2-precoated microplate. Briefly, 0.1 μg/well of SARS-CoV-2 spike protein with a streptavidin tag or the horseradish peroxidase (HRP)-conjugated recombinant SARS-CoV-2 RBD fragment was incubated with ATRA at 37°C for 30 min. DMSO was used as a negative control, and the hACE2 antibody was used as a positive control. The mixture was then added to the precoated microplate at 37°C for 15 min, and the unbound protein was removed by three washes with the wash buffer provided with the kit. For the spike binding ELISA, HRP-labeled streptavidin (Beyotime catalog no. A0303) was added at a dilution of 1:4,000, and the mixture was incubated at 37°C for 45 min. After four washing steps, TMB (3,3′,5,5′-tetramethylbenzidine) solution was added to the microplate, and the mixture was incubated at room temperature. Then, the stop solution was added, and the absorbance was detected at 450 nm.

### Coimmunoprecipitation assay.

Purified human ACE2 protein with a Flag tag and purified spike protein with Twin-Strep-tag were used in the coimmunoprecipitation assay. Briefly, 2 μg of spike protein was preincubated with or without ATRA at 37°C for 30 min. The mixture was then added to 2 μg human ACE2 protein at 4°C for 2 h. About 5 μL anti-Flag antibody (Cell Signaling Technology catalog no. 8146S) was added, and the mixture was incubated for 2 h at 4°C. The mixture was incubated with 20 μL prewashed protein A/G agarose (Thermo catalog no. 20422) at 4°C for 5 h with gentle mixing. The immunoprecipitated complexes were collected by centrifugation at 3,000 × *g* for 2 min and washed with 500 μL immunoprecipitation (IP) lysis buffer (Thermo catalog no. 87787) 6 times. The pellet was resuspended in 30 μL protein loading buffer and heated at 100°C for 10 min. Samples were then subjected to Western blot analysis.

### Cryo-EM sample preparation and data collection.

ATRA, dissolved in DMSO at a concentration of 100 mM, was added to the purified SARS-CoV-2 spike protein (approximately 0.5 mg/mL), mixed thoroughly to a final concentration of 40 μM, and incubated at room temperature for 30 min. An identical volume of DMSO was added to another copy of the spike protein. The protein was then cross-linked with 0.015% glutaraldehyde on ice for 20 min. Tris stock buffer (2 M [pH 7.5]) was added to stop the cross-linking reaction. Four microliters of the spike-DMSO or spike-ATRA sample was applied onto a freshly glow-discharged Quantifoil R1.2/1.3 carbon grid. The grids were blotted for 10 s at 100% humidity and 8°C before flash freezing in liquid ethane with Vitrobot MarkIV 20 (Thermo Fisher Scientific). Data were acquired on a Titan Krios electron microscope (FEI Company) equipped with a field emission gun operated at 300 kV and equipped with a Gatan K3 Summit direct detector. Data were collected in superresolution mode at a nominal magnification of 29,000× with a virtual pixel size of 0.97 Å. A total of 4,049 micrographs of spike-ATRA and 2,089 micrographs of spike-DMSO were collected. The defocus range was set between −0.5 and −1.5 μm. A full description of the cryo-EM data collection parameters can be found in Table S2.

### Cryo-EM data processing.

The beam-induced motion of each micrograph was corrected with MotionCor2 (48). The defocus value was estimated with Gctf (49). Particles were picked automatically with Relion 3.0 software (50). After 5 rounds of reference-free 2D classification, 97,223 and 98,583 particles, respectively, for spike-ATRA and spike-DMSO samples with well-organized features were selected for 3D analysis. Previously reported spike cryo-EM maps in open, closed, and intermediate states (EMD-11203, EMD-11205 and EMD-11206, respectively [30]) were used as the initial models for heterogeneous 3D classifications and refinements in cryoSPARC without applying any symmetry (C1). Then, the spike-ATRA complex particles in the closed state were selected and used to perform nonuniform refinement and local refinement in cryoSPARC, with C3 symmetry imposed. Reported resolutions were based on the “gold standard” Fourier shell correlation (FSC) of the 0.143 criterion (51). The directional FSC curves were calculated by using the Remote 3DFSC Processing server (https://3dfsc.salk.edu) (52). The local resolution of the cryo-EM density maps was calculated by using cryoSPARC.

### Cryo-EM model building and analysis.

UCSF Chimera (53) and Coot (54) were used to fit closed spike trimer atomic models (PDB ID 6VXX [32]) into the C3 cryo-EM map. The model was then manually rebuilt in Coot. N-linked glycans were hand-built into the density where visible. The model for ATRA was manually fit into the additional narrow density at the interfaces of adjacent RBDs all in the “down” conformation. This model for the spike-ATRA complex was then refined in real-space by using Phenix (55).

### Quantification and statistical analysis.

Descriptive statistics are provided in the figure legends. Analyses of independent data were performed by using the unpaired *t* test. Statistical analyses were carried out using GraphPad Prism 8.0. *P* values of <0.05 were considered significant.

### Data availability.

All data are available in the main text or the supplemental material. The electron microscopy (EM) map has been deposited in the Electron Microscopy Data Bank (EMDB) under accession no. EMD-33600, and the accompanying atomic coordinates have been deposited in the Protein Data Bank (PDB) under accession no. 7Y42.
