# Supplementary material for: A Retinol Derivative Inhibits SARS-CoV-2 Infection by Interrupting Spike-Mediated Cellular Entry
Source: mBio. 2022 Jul 13;13(4):e01485-22. doi: 10.1128/mbio.01485-22 (PMC9426596; doi:10.1128/mbio.01485-22)
Supplement: TABLE S1 [file mbio.01485-22-s0008.docx]

**Table S1.** **Primers for qRT-PCR.**

| **The primers for qRT-PCR** | **Forward primer** | **Reverse primer** |
| --- | --- | --- |
| SARS-CoV-2 *ORF1ab* | AGAAGATTGGTTAGATGATGATAGT | TTCCATCTCTAATTGAGGTTGAACC |
| IAV H1N1 *NP* | GACGATGCAACGGCTGGTCTG | ACCATTGTTCCAACTCCTTT |
| human *GAPDH* | AGCCTCAAGATCATCAGCAATG | ATGGACTGTGGTCATGAGTCCTT |
