# Supplementary figures and images for: A Retinol Derivative Inhibits SARS-CoV-2 Infection by Interrupting Spike-Mediated Cellular Entry
Source: mBio. 2022 Jul 13;13(4):e01485-22. doi: 10.1128/mbio.01485-22 (PMC9426596; doi:10.1128/mbio.01485-22)

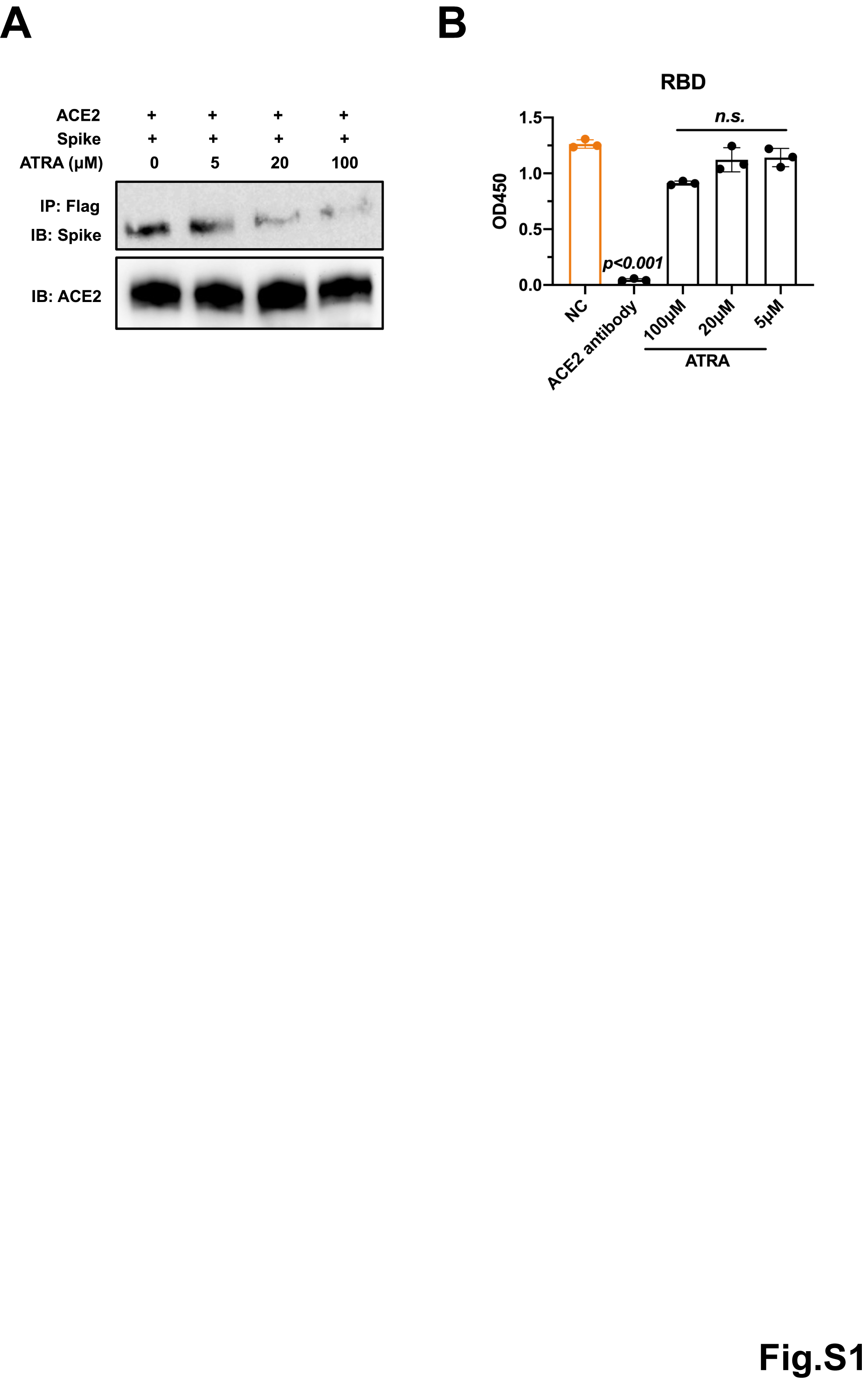

Supplement: FIG S1 [file mbio.01485-22-s0001.tif]

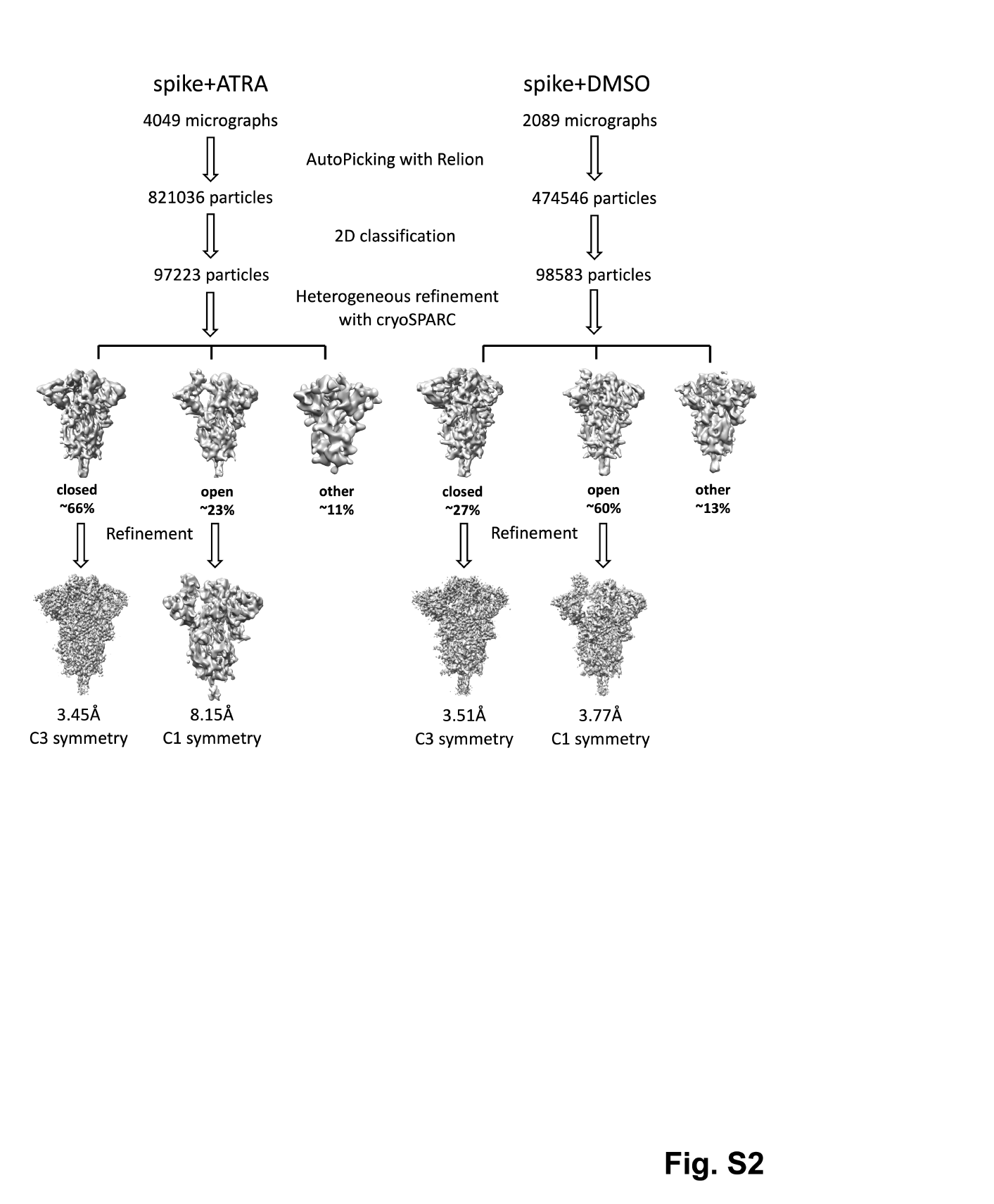

Supplement: FIG S2 [file mbio.01485-22-s0002.tif]

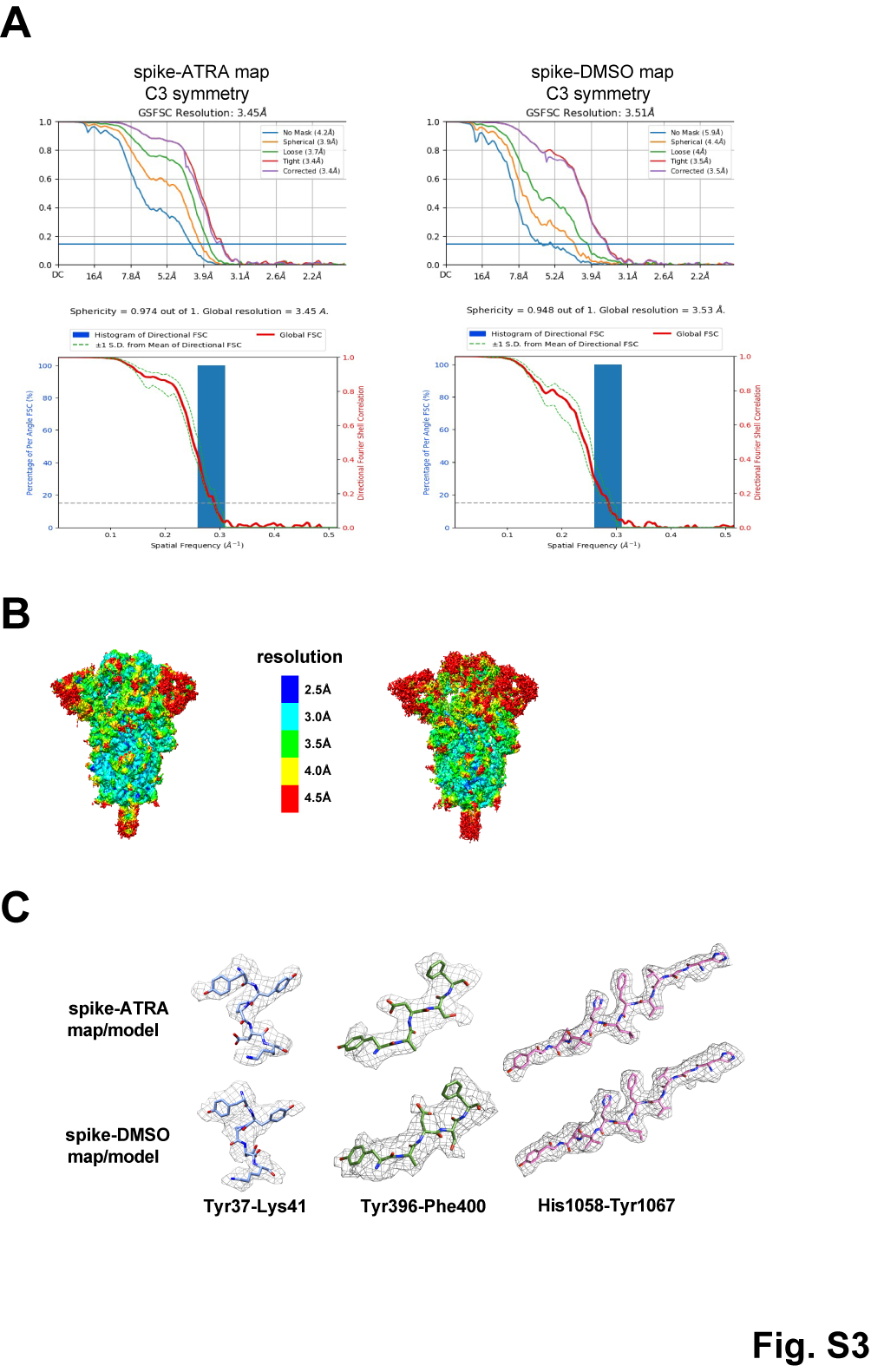

Supplement: FIG S3 [file mbio.01485-22-s0003.tif]

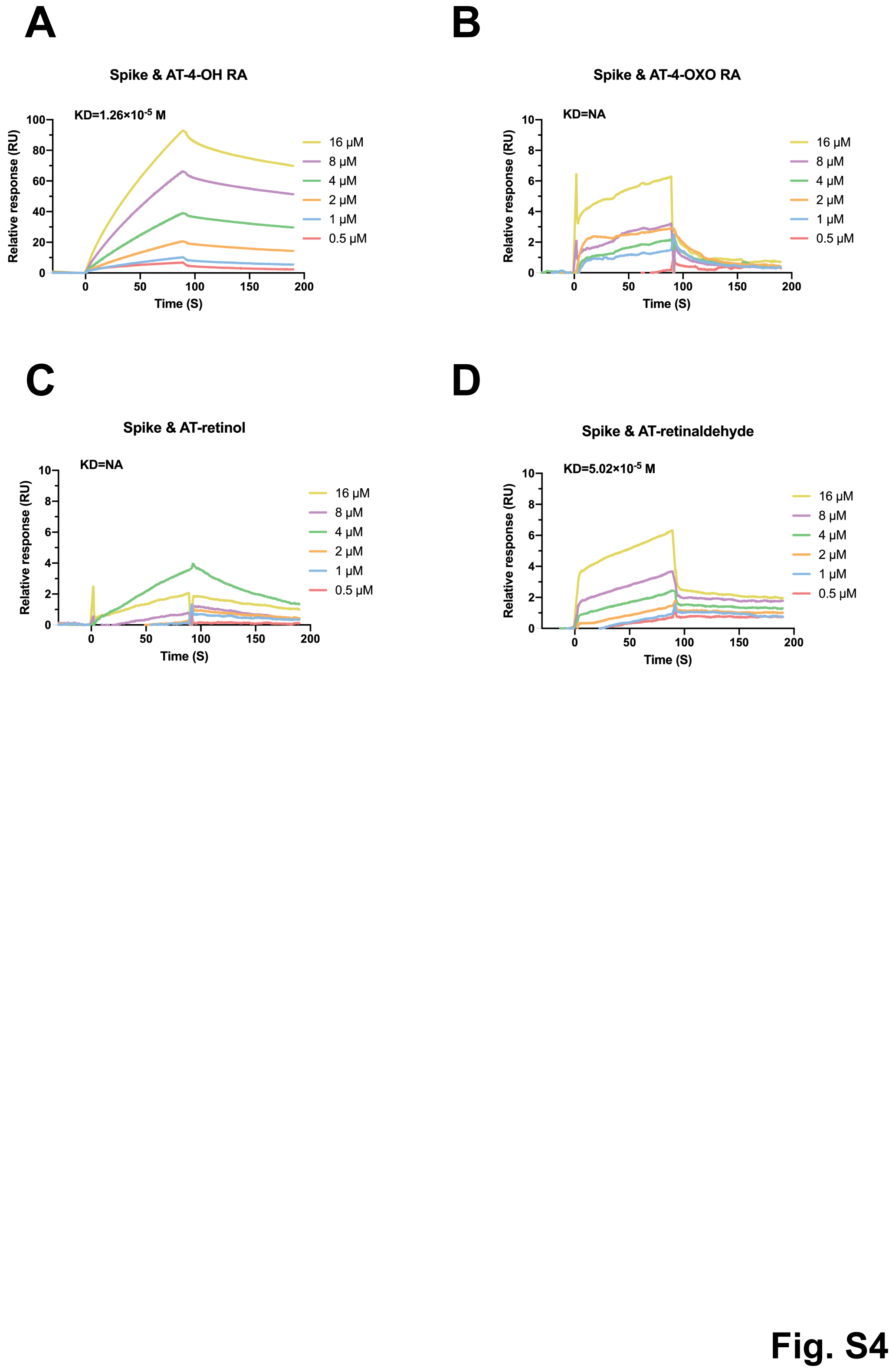

Supplement: FIG S4 [file mbio.01485-22-s0004.tif]

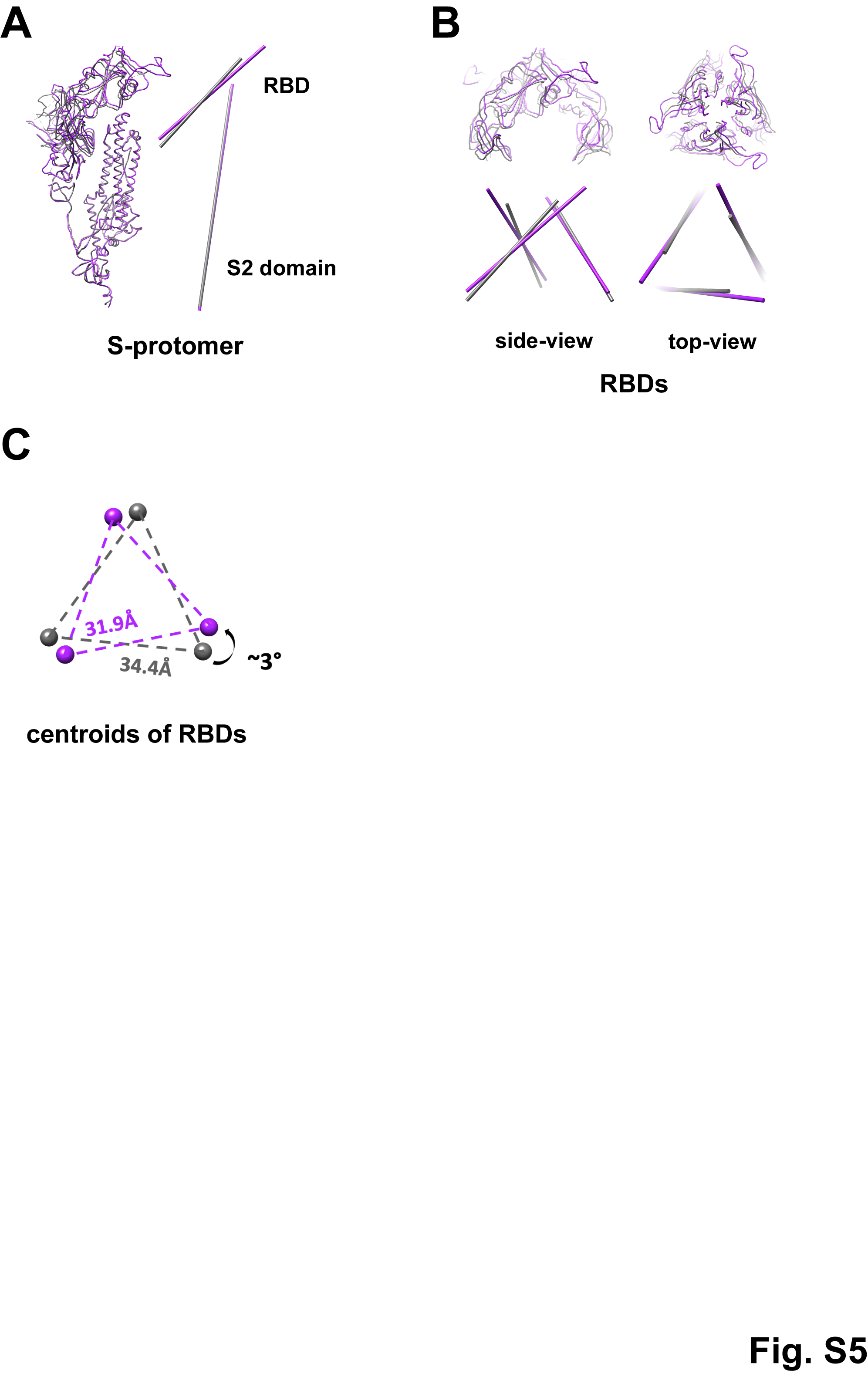

Supplement: FIG S5 [file mbio.01485-22-s0005.tif]

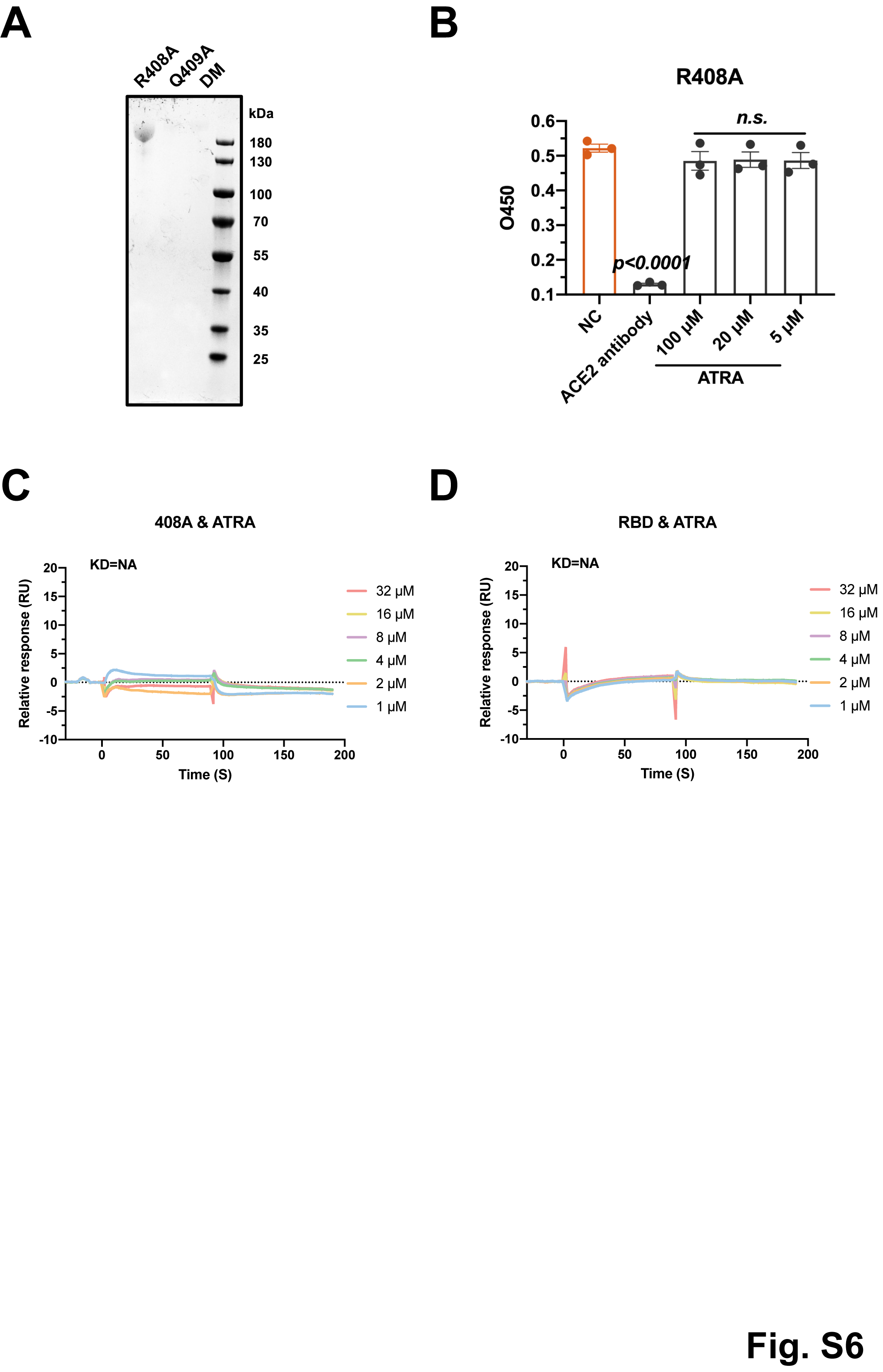

Supplement: FIG S6 [file mbio.01485-22-s0006.tif]

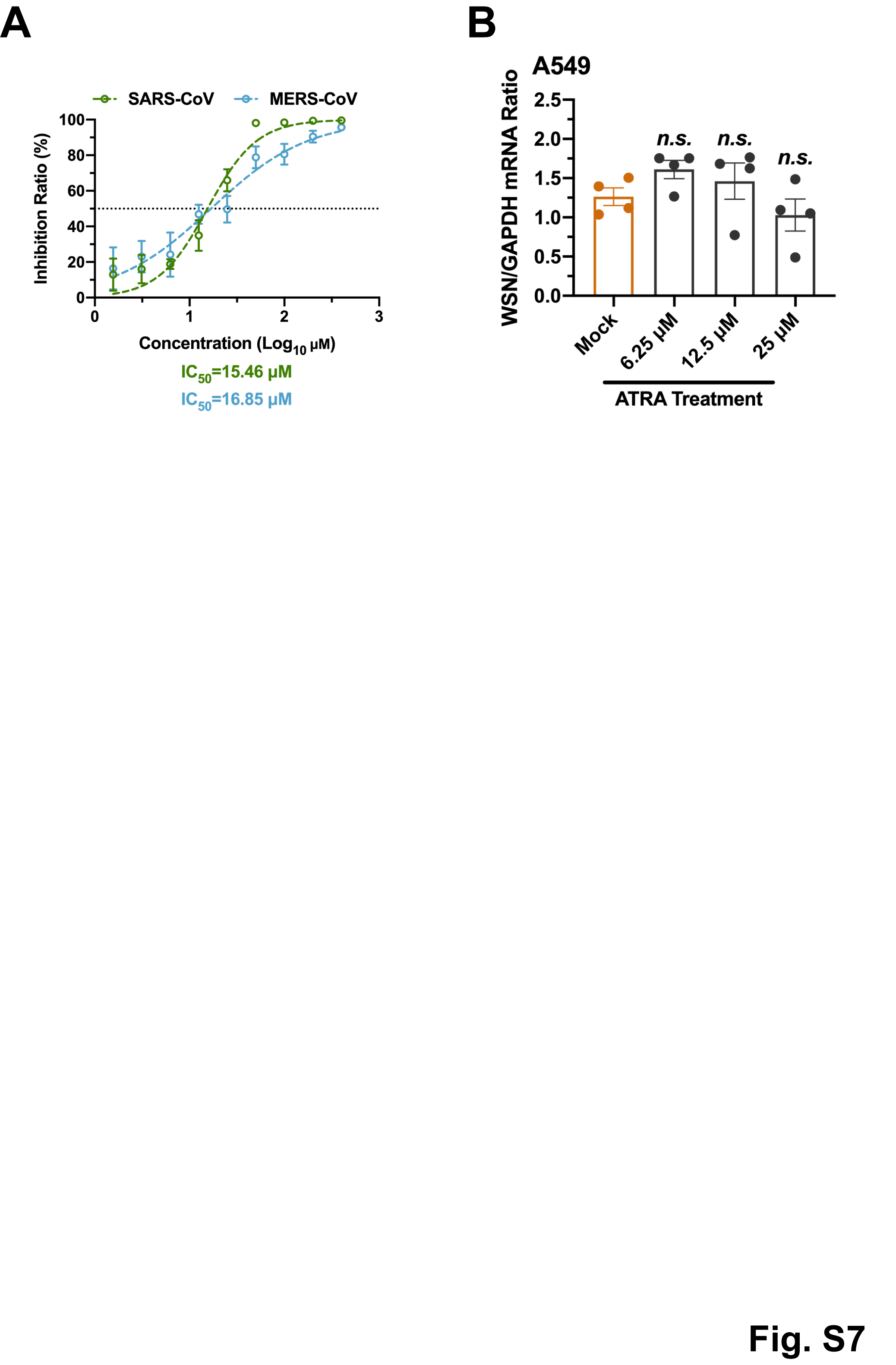

Supplement: FIG S7 [file mbio.01485-22-s0007.tif]
