# Supplementary material for: A Retinol Derivative Inhibits SARS-CoV-2 Infection by Interrupting Spike-Mediated Cellular Entry
Source: mBio. 2022 Jul 13;13(4):e01485-22. doi: 10.1128/mbio.01485-22 (PMC9426596; doi:10.1128/mbio.01485-22)
Supplement: TABLE S2 [file mbio.01485-22-s0009.docx]

**Table S2.** **Cryo-EM data collection, refinement and validation statistics.**

|  | **ATRA-treated spike** | **DMSO-treated spike** |
| --- | --- | --- |
| **Data collection and processing** |  |  |
| Magnification | 29000 | 29000 |
| Voltage (kV) | 300 | 300 |
| Electron exposure (e^–^/Å^2^) | 50 | 50 |
| Frames per exposure | 32 | 32 |
| Defocus range (μm) | -0.5 to -1.5 | -0.5 to -1.5 |
| Pixel size (Å) | 0.97 | 0.97 |
| Micrographs collected | 4049 | 2089 |
| Particles final | 66436 | 26550 |
| Symmetry imposed | C3 | C3 |
| Map sharpening B-factor (Å^2^) | -115.3 | -90.5 |
| Resolution at 0.143 FSC (Å) | 3.45 | 3.51 |
| **Refinement** |  |  |
| Model composition |  |  |
| Protein residues | 2904 | 2916 |
| Glycans | 27 | 27 |
| Ligands (ATRA) | 3 | -- |
| R.M.S. deviations |  |  |
| Bond lengths (Å) | 0.006 | 0.007 |
| Bond angles (°) | 0.796 | 0.821 |
| Mean B-factors (Å^2^) |  |  |
| Amino acids | 71.80 | 164.04 |
| Ligands | 119.56 | 189.69 |
| **Validation** |  |  |
| Ramachandran |  |  |
| Favored (%) | 90.48 | 92.48 |
| Allowed (%) | 9.52 | 7.52 |
| Outliers (%) | 0.00 | 0.00 |
| Rotamer outliers (%) | 0.59 | 0.70 |
| Clash score | 11.69 | 16.36 |
| C-beta outliers (%) | 0.00 | 0.00 |
| CaBLAM outliers (%) | 4.18 | 2.71 |
| CC (mask) | 0.81 | 0.80 |
| MolProbity score | 2.12 | 2.18 |
